# Metacognition, Mind Wandering, and Cognitive Flexibility: Understanding Creativity

**DOI:** 10.3390/jintelligence10030069

**Published:** 2022-09-16

**Authors:** David D. Preiss

**Affiliations:** Escuela de Psicología, Facultad de Ciencias Sociales, Pontificia Universidad Católica de Chile, Av. Vicuña Mackenna 4860, Macul, Santiago 7820436, Chile; davidpreiss@uc.cl

**Keywords:** creativity, metacognition, mind wandering

## Abstract

The goal of this article is to review work on mind wandering, metacognition and creativity in order to consider their relationship with cognitive flexibility. I introduce a model of the role that mind wandering and metacognition have in the generation and exploration of novel ideas and products in the creative process. I argue that managing the interaction between metacognition and mind wandering is the main role of cognitive flexibility in creativity. Furthermore, I claim that balancing the influence of metacognition during the generation and exploration of pre-inventive structures is a quintessential part of creativity, probably in almost any domain. Thus, I advance a general framework that can be applied to understanding how creators monitor and think about their own cognition when they engage in the generation and exploration of ideas. Additionally, I discuss the evolution of controlled and spontaneous cognition and metacognitive judgements during the development of a creative person.

## 1. Introduction

The goal of this article is to review work on mind wandering, metacognition, and creativity in order to consider their relationship with cognitive flexibility. Additionally, the article presents a framework to understand the role that metacognition plays in the development of a creative person. The literature addressing the relationship between creativity and metacognition is not abundant, although the topic gained a growing attention in the last decade, especially among researchers working on mind wandering and creativity (Fox and Christoff 2014; Kaufman and Beghetto 2013; Preiss et al. 2016, 2020a). Kaufman and Beghetto defined creative metacognition as “a combination of creative self-knowledge (knowing one’s own creative strengths and limitations, both within a domain and as a general trait) and contextual knowledge (knowing when, where, how, and why to be creative)” (Kaufman et al. 2016, p. 260; see also Kaufman and Beghetto 2013). Here, I want to complement this definition by adding to self-knowledge and contextual knowledge the metacognitive processes associated with the generative and exploratory stages of creativity. I argue that managing the interaction between metacognition and mind wandering is the main role of cognitive flexibility in creativity. Furthermore, I claim that balancing the influence of metacognition during the generation and exploration of pre-inventive structures is a quintessential part of creativity, probably in almost any domain. Thus, I advance a general framework that can be applied to understanding how creators monitor and think about their own cognition when they engage in the generation and exploration of ideas. It is worth noting that this framework only applies to one specific case of creative production, specifically those cases involving generation and exploration of ideas. It must be noted that creativity involves other phenomena, such as those involving trial-and-error or serendipity, which do not fit this conception very well. Yet, my focus on the generation and exploration of creative ideas originates in my interest in connecting the creative process with cognitive flexibility.

As noted by the GENEPLORE model (Finke 1996; Finke et al. 1992), there are two phases in creativity: a generative phase and an exploratory phase. Other models of creativity consider more stages or processes. To illustrate, Mumford et al. proposed eight different processes to explain creativity (Mumford et al. 1991, 2018). Yet, for the argument advanced here, the GENEPLORE model is a better fit. According to this model, the generative phase involves the creation of representations, and the exploratory phase involves the evaluation of those representations in search of a meaningful and definitive product. These two processes can also be defined using the concepts of the blind-variation and selective-retention theory of creativity (Simonton 2022). In short, the generative phase involves blind variation, and the exploratory phase involves selective retention. Finke et al. (Finke 1996; Finke et al. 1992) labelled the representations generated during the generative phase as pre-inventive structures since they implicate a discovery process. They noticed that the two phases—generative and exploratory—are not sequential but interactive, although they are limited by mental ability. Predictably, individuals with higher intelligence display a larger ability to recognize and properly assess their own creativity than their peers (Karwowski et al. 2020). Understanding how metacognition interacts with these two stages of creativity is instrumental to investigating creative processes not only in laboratory conditions, but also in real life (Cosmelli and Preiss 2014). Here, I argue that metacognition is a key component of cognitive flexibility during the creative process as well.

In brief, I propose that, during the generative stage, metacognition plays a balancing role in attention regulation, specifically in mind wandering. Additionally, during the exploratory stage, metacognition shapes self-judgment of creative products. To produce novelty, creators alternate between controlled and spontaneous cognition. This idea is consistent with established findings in creativity research suggesting that creative individuals can focus or defocus their attention depending on the nature of the task (Martindale 1999; Martindale and Hasenfus 1978). This ability to alternate between controlled and spontaneous cognition is central to cognitive flexibility. As expertise in a domain develops, creators’ use of metacognition during the two stages of the creative process changes. At the highest levels of creative expertise, creators develop a metacognitive strategic approach to mind wandering, and they develop a disposition to mindful mind wandering (Preiss and Cosmelli 2017).

As creativity develops, individuals not only learn how to monitor their episodes of mind wandering, but also how to make the most of its constructive dimension during idea generation. In addition, the exploratory phase increases in complexity and scope. Particularly, as the creative process becomes temporally extended, the nature of metacognitive judgments also shifts. Whilst at the earliest stages of creative development metacognitive judgements are absent or localized and focused on a specific task or product, at the most advanced phases of creativity, metacognitive judgements grow to progressively take into consideration an entire oeuvre of work. As they are part of a whole system of self-beliefs, these metacognitive judgments about the quality of a creator’s own work have relevant implications for the development of a creator’s self (Beghetto and Karwowski 2017), and they become intertwined with what is known as networks of enterprise (Gruber 1988; Gruber and Wallace 2001).

## 2. Mind Wandering, Metacognition, and Creativity

Mind wandering is a prime example of the stream of our conscious experience as well as of the continuous shifts of our attention (Smallwood and Schooler 2015). People mind-wander when self-generated thinking triggers a shift of their attentional focus from the task they are performing to their own feelings and thoughts (Smallwood and Schooler 2015). Although it is a natural inclination of the human mind, mind wandering has a negative impact on activities demanding sustained attention (Mrazek et al. 2012; Smallwood et al. 2007). Additionally, poor executive control triggers mind wandering during demanding tasks (Kane and McVay 2012; McVay and Kane 2012). Still, some research suggests that mind wandering plays an important function during incubation and idea generation of pre-inventive structures. Episodes of mind wandering during incubation are correlated with the individuals’ ability to solve creative problems (Baird et al. 2012). By reducing cognitive control, mind wandering can facilitate the emergence of remote associations. Thus, it makes more likely the generation of unexpected solutions during creative problem-solving. Additionally, deliberate mind wandering, as the one used during guided imagery (Finke 1996; Singer and Barrios 2009), can be used to strengthen creativity. To illustrate, poets engage deliberatively on mind wandering during creative writing (Preiss et al. 2020b; Preiss and Cosmelli 2017). It is worth noting that trait mind wandering is also related to creativity. For instance, it predicts verbal creativity over the contribution of fluid intelligence, reading difficulties and declarative knowledge of metacognitive strategies in tertiary-level students (Preiss et al. 2016).

Mind wandering can be regulated by meta-awareness. Meta-awareness has been defined as “one’s explicit knowledge of the current contents of thought” (Schooler et al. 2011, p. 321). Meta-awareness makes possible to identify mind wandering episodes and facilitate reengagement with the primary task or with the activities that help people to have more control of their activity. Although it is connected with meta-awareness, metacognition is not the same. Metacognitive activity may involve awareness, yet it may happen without awareness as well (Schooler 2002). Metacognition involves cognition related to one’s own cognitive processing, including both metacognitive knowledge and metacognitive experiences (Flavell 1979). A close concept, coined by research on clinical neuropsychology, is that of executive function. It refers to the higher-order cognitive processes allowing a more flexible and adaptive behaviour. Both are closely related (Roebers 2017).

Efklides distinguishes between three dimensions of metacognition: metacognitive knowledge, metacognitive experiences, and metacognitive abilities (Efklides 2006, 2008). According to Efklides, metacognitive knowledge is declarative knowledge and includes information about persons, strategies, tasks, and goals. Metacognitive experiences refer to the awareness and feelings a person has when he or she is implementing a task. Finally, metacognitive abilities are those skills individuals use to control cognition and include several types of metacognitive strategies including planning, regulation, and evaluation, among others. Efklides’ model is helpful to think about the relationship between creativity and metacognition. I think that these three dimensions of metacognition are engaged differently during the generative and exploratory stages of the creative process. Metacognitive experiences are, probably, more important in the generative stage than in the exploratory stage since they are helpful for identifying attentional shifts and regulating them during idea generation, particularly mind wandering. Metacognitive abilities might be triggered during both generation and exploration of pre-inventive structures by metacognitive experiences and metacognitive knowledge. Metacognitive knowledge might be more influential in the exploratory stages of creativity as creators must apply their knowledge to assess how appropriate a product is, what strategies are more useful to improve it, and whether their skills are up to the task or some collaboration from other individuals is needed. 

Based on the evidence currently available, it is possible to think of three possible relationships between the creative process and metacognition. First, metacognition might limit creativity, especially during the generative stage. Spontaneous cognition during constructive mind wandering is associated with creativity (Preiss et al. 2020a). Creativity involves defocusing attention and cognitive disinhibition (Martindale 1999). On the other hand, metacognition requires cognitive control and might set limits for creativity by not giving enough room for the mind to wander. Therefore, metacognition might stop idea generation by constraining spontaneous new associations, which are essential to the creative process. If a creator controls spontaneous thought, it makes the generation of some unexpected original associations less probable. Additionally, metacognition might produce perseveration errors that derail insight. As it is known, the whole experience of insight is based on the premise that solutions arrive when the problem solver stops perseverating in the same strategy (see chapters in Davidson and Sternberg 1995). Therefore, it is plausible to hypothesize that there is a trade-off between metacognition and creativity. On the contrary, it is also plausible to hypothesize that metacognition facilitates creativity. Metacognition might be necessary for creativity, especially during the exploratory stage (Fox and Christoff 2014) and the evaluation of creative products (Armbruster 1989; Puryear 2015). Creativity involves the generation of a novel product that is also appropriate (Kaufman and Beghetto 2009). Therefore, finalizing a novel product involves the ability to assess pertinence in addition to originality (Runco et al. 2005; Runco and Charles 1993). The mere generation of bizarre products or the fact that they were made by a creative personality do not turn a product into a creative one, especially in fields where functionality and correctness are relevant, such as in the STEM domains. Consequently, appropriateness is part and parcel of the assessment of creativity, both in research and in real-life endeavours. Psychometric studies of creativity assess appropriateness in restricted domains whereas real-life creativity involves a systematic assessment of creativity outputs by the gatekeepers in the field such as peer reviewers, editors, critics, and so on (Csikszentmihalyi 1996, 2014). From the point of view of a creator, revision of the appropriateness of pre-inventive structures requires a great deal of cognitive effort and metacognitive regulation. Thus, on the one hand, there may exist trade-offs between metacognition and creativity during generation of ideas and, on the other hand, there may exist metacognitive facilitation of creativity during the exploration of ideas. A third alternative is that creativity requires a continuous balancing act between spontaneous and controlled cognition, and therefore metacognition’s impact on the creative process changes across different levels of creative expertise. That is, creativity requires cognitive flexibility.

## 3. Cognitive Flexibility and the Wandering Mind

According to Yu et al. (2019), cognitive flexibility can be understood as a meta-competence. Specifically, it is instrumental for adaptive performance, which should be responsive to novel challenges in evolving environments. According to the authors, cognitive flexibility differs from human intelligence because the latter is a static measure of performance whereas the former tests how individuals shift their cognition to respond to novel challenges in problem-solving. As Beckmann (2014) notices, cognitive flexibility involves the ability to deal with novelty, an attribute that is shared with some definitions of intelligence such as the construct of fluid intelligence. Yet, conventional tests of intelligence are not tests of cognitive flexibility since they do not introduce novelty during the test situation. Thus, tests of cognitive flexibility are in an intermediate zone between static and dynamic assessments of ability. Beckman argues:

Within a Vygotskian framework, I would argue that learning tests aim at the identification of the “zone of proximal development”; cognitive flexibility tests aim at the plasticity of the “zone of current development.” One might then speculate that sufficient levels of plasticity in maintaining high levels of (cognitive) performance indicate adequate consolidation of current developmental achievements, which is one precondition for affording a wider horizon when venturing into the zone of proximal development.

(p. 320)

What allows for a successful completion of the creative process is the ability of individuals to monitor their attentional focus during the creative process. They must be able to shift from spontaneous to controlled cognition and back to allow room for the generation of new associations and implement a thorough process of revision. These processes are certainly interactive. 

What is, then, the relationship between cognitive flexibility, metacognition, and creativity? I propose that cognitive flexibility during the creative process involves a strategic use of mind wandering by means of metacognitive regulation. First, spontaneous idea generation might be inhibited by metacognitive regulation. Yet, metacognitive regulation is necessary during creative exploration or evaluation (Fox and Christoff 2014). Creators need to develop the ability to attenuate their level of metacognitive control during the generation of ideas but strengthen metacognitive regulation when they evaluate their creative products. Therefore, creative achievement rests on cognitive flexibility and the capacity to manage, allocate, and distribute attentional resources during extended problem-solving under the guidance of metacognitive strategies. From the point of view of the two attributes of creativity, originality and appropriateness or fit (Runco 2004; Runco et al. 2005; Runco and Charles 1993), mind wandering feeds originality whereas metacognition feeds appropriateness. Since novelty rests on new associations, mind wandering creates space for novelty to emerge. In turn, metacognition is instrumental to the deliberate process of revising their level of appropriateness or fit with the domain. These processes are not necessarily localized in time. In real life, the shift between idea generation and revision is continuous and capitalizes on a permanent process of attentional management. 

**Figure 1:**
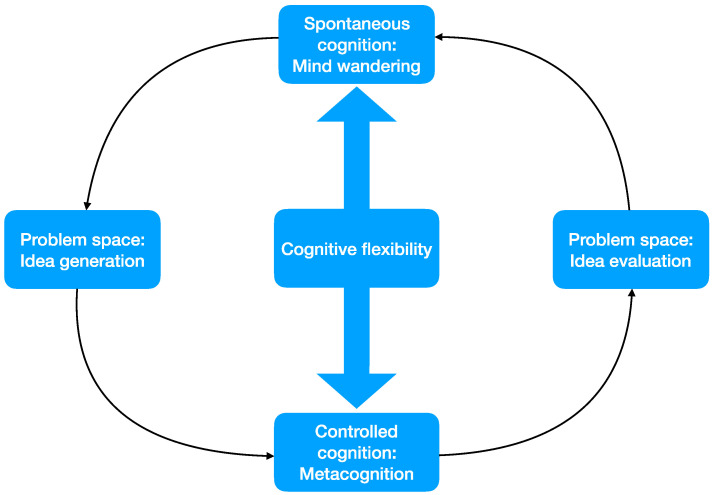
Cognitive flexibility and the creative process.

Figure 1 summarizes this process. As already mentioned, the creative process involves finding an idea and assessing its appropriateness (Finke 1996; Finke et al. 1992). That is, the creative process involves dealing with two problem spaces: idea generation and idea evaluation. Cognitive flexibility helps to alternate between spontaneous and controlled cognition. During idea generation, spontaneous cognition allows the generation of new associations. During idea evaluation, controlled cognition is instrumental to implementing the process of revision. This idea is consistent with the variation–selection models of creativity, such as the blind-variation and selective-retention creativity theory (Campbell 1960), and particularly more recent developments of this theory such as the one advanced by Simonton (2022). As noted above, idea generation can also be understood as blind variation, and idea evaluation as selective retention. The process ends when the creator decides that the product is completed or the passing of time or a deadline stops further iterations. As creativity evolves from the initial stages of creativity to the more advanced ones, the shifts between spontaneous and controlled cognition progress towards an integrated and strategic use of cognitive flexibility.

## 4. Development of a Creative Person

In order to understand the growth of creativity, Kaufman and Beghetto (2009) proposed a developmental model of creativity that distinguishes between four levels of creativity: mini-C, little-C, Pro-C, Big-C. In the first level, the mini-C level of creativity, individuals produce something that is mostly meaningful to them. Thus, the mini-C level of creativity is related to those creative insights experienced during the learning process. It is represented by emerging mental constructions that are typical in young children but that are also present in adults. Mini-C creativity is involved in what may be considered as first-stage pre-inventive structures. Little-C creativity involves a product that triggers a positive assessment from other persons (such as peers or teachers), yet this valuation is not a professional one. It is the kind of creativity observed in everyday creativity. According to Kaufman and Beghetto, the Pro-C level of creativity involves professional contributions in a domain and, therefore, expert assessment. This level of creativity is exhibited by individuals who, after undergoing a significant amount of practice, have been able to create a product that is deemed as original and appropriate by the gatekeepers of a specific field. The authors propose that the Big-C level of creativity is a significant step beyond the Pro-C level and considers the production of an entire body of work, which has made a difference in a cultural field. Thus, Big-C creativity is eminent creativity. It is worth noting that although the authors see this model “as representing a developmental trajectory of creativity in a person’s life” (Kaufman and Beghetto 2009, p. 6), they do not think that it is necessary to pass through each of these levels of creativity to become an eminent creator. 

Next, I will complement this theory with the previous discussion about cognitive flexibility. At the mini-C or little-C levels, and during the generative stage of the creative process, spontaneous cognition is more dominant than controlled cognition. The main activity driving mini-C creativity is generation or blind variation. As contributions at this level have mostly personal value, they involve idea generation with relatively limited attention regulation and metacognitive control. Thus, spontaneity plays an important role in idea generation whereas metacognition plays a limited and localized role in the evaluation of creative products. Still, as children are schooled, metacognition becomes slightly more important in little-C than in mini-c creativity. In the Pro-C and Big-C levels, controlled cognitive processes become increasingly more important than the spontaneous ones. Attention regulation as well as metacognitive assessment become interwoven with the creative process. Metacognitive judgments are not self-generated. They incorporate the dominant ideas from the field about appropriateness and, probably, the standards set by the gatekeepers of the field. To illustrate, scientists become concerned with citations and peer reviews in addition to their self-assessment of their work.

Thus, in its initial stages, the core activity in the creative process involves mostly tinkering with ideas and products. In turn, this implicates improvisation. When improvising, individuals actualize their creative potential spontaneously, as illustrated by pretended play (Russ 2020). Indeed, one of the main attributes of children’s creativity is its unprompted nature. Unsurprisingly, it is commonplace for eminent creators to appeal to childhood’s innocence when they want to reclaim the original properties of the creative process. Still, spontaneity is not exclusive of childhood, as demonstrated by its importance in activities as disparate as jazz or teaching (Sawyer 1992, 2004). As children enter school and progress across its grades, they are provided with an early education in different academic and artistic disciplines. Specifically, children creativity is “disciplined” and constrained by their teacher expectations about what is right and what is wrong, probably through a Vygotskian process of internalization (Vygotsky 1978). Thus, spontaneity in idea generation is educated and regulated with mixed results. As it is known, schools are one of the main drivers of cognitive growth in childhood and adolescence (Ceci 1991; Cole 2005; Olson 1996; Preiss 2020; Preiss and Sternberg 2005). Executive function, intelligence and metacognition are developed in school, and all of them start to play a relevant role in the consolidation of creative skills. Therefore, high school students are able to capitalize on the creative potential of mind wandering at the highest levels of attentional capacity and metacognition (Preiss et al. 2019). Unfortunately, schools are not particularly eager to foster creative skills and, therefore, many educational reformers and psychologists have criticized that they fail in developing abilities which have creative value in real life (Bruner 1996; Cole 2005; Sternberg 2002). 

After finishing school, some individuals may transition to become experts in a field and work professionally in disciplines requiring creativity. Development of expertise in a domain requires full-time involvement and repeated practice under the guidance of a mentor (Ericsson et al. 1993; Ericsson and Charness 1994; Hass and Weisberg 2015), although there may be exceptions to this path, particularly among the most talented individuals in a domain. This is particularly true of those disciplines where individuals produce eminent contributions early. As noted above, not all eminent creators pass through the four levels of the 4-C model. Furthermore, although Kaufman and Beghetto’s notion of Pro-C is consistent with the expertise models of creativity, the authors recognize the limitations of their approach. Not only the amount of time necessary to reach professional proficiency in a domain does not always adjust to the 10-year rule, but also the activities involved in the process do not always involve repeated practice (Gardner 1993). They can also involve experimentation, idea generation, or blind variation. As they become more familiarized with a discipline, individuals learn how to assess their creative output and internalize the expectations of their professional fields. Without proper evaluation, which in turn requires metacognition, there is no way to produce a contribution deemed appropriate by the gatekeepers in the field and survive professionally (Csikszentmihalyi 1996, 2014). Later, within that privileged subset, just a few individuals reach eminence within their fields. These individuals are freed from many of the restrictions that experts face when they are in the initial stage of their professional careers (in academic jargon, they get tenure). Therefore, their approach to the creative process is now less constrained by the initial restrictions of the field. Of course, there are instances where some individuals revolutionize their fields by going against these restrictions when they are very young in their careers. That said, a Rimbaud is more likely to happen in poetry than in biomedicine. This is true not only for poetry but also for other disciplines such as mathematics (Simonton 2018).

Let us explore this sequence more deeply. During an initial state, especially in childhood, individuals explore different domains. Generation of ideas is unconstrained by domain knowledge. Evaluation of achievement is mainly based on a personal perspective and conducted for specific tasks or activities. Creativity has an expressive nature, mostly. Children can produce drawings and stories, find that they are creative or beautiful, and look for approval within their immediate relationships. Furthermore, they can be impressed by their own ability to produce novelty and generate surprise. However, they have not yet developed the ability to judge or consider the appropriateness of their ideas. This stage of innocence is lost after they enter formal schooling. As children are exposed to various disciplines, they learn that domains have rules and that those rules set limits to what can be performed in a domain. As some teachers like to say, children come to school to learn, not to play. Certainly, the dissociation between schooling and play is negative for the development of creativity. Unfortunately, during the current century, preschool education has also been affected by this (Hirsh-Pasek et al. 2008). As children are schooled, adult feedback becomes increasingly important and integrated with their own self-appraisal. Thus, children learn what is appropriate in a discipline, many times at the cost of spontaneity. Their experiences with the feedback of others play a relevant role in preparing individuals to more sophisticated forms of training, but also have an impact on their self-esteem and feelings of competency in a domain. Furthermore, access to mentors during adolescence is very relevant in those domains where contributions are made very early on in the life of an individual. 

For those individuals who decide and have access to the opportunities allowing them to embark on a professional route, metacognition starts to play a deeper role. In many domains, professional training requires specialized instruction and deliberate practice (Ericsson et al. 1993; Ericsson and Charness 1994). However, deliberate practice is not enough to explain professional creativity. One of the most important metacognitive abilities professional creators must develop is the capacity to mindfully mind-wander, which is the ability to identify and capitalize on mind wandering during long-term creative enterprises (Preiss and Cosmelli 2017). That is, creators start to recognize the role that spontaneous cognition plays in the generative stage of creativity. Their meta-awareness of their mind-wandering process allows them to put spontaneous cognition at the service of the creative process and use controlled cognition in a manner that does not interfere with idea generation. It should not be implied, however, that creators do not experience situations where ideas are spontaneously generated. What they are now capable of is recognizing that these events are part of extended creative endeavours. Furthermore, if they want to survive in their field, experts must be able to produce contributions that are relevant to their domain and must deal with the gatekeepers of their field. As it is known, disseminating a new idea into a domain involves not only idea generation, but also the ability to choose ideas which are worth investing in (Sternberg and Lubart 1996). Therefore, metacognition is also employed in the process of selling an idea during interaction with gatekeepers, such as in peer review in science or in the interactions with arts and literary critics. As some experts reach higher levels of eminence, they get involved in broader networks of enterprise (Gruber and Wallace 2001). Some of them become polymaths and transfer their creative skills across domains (Root-Bernstein and Root-Bernstein 2004). Additionally, some creators become gatekeepers in their fields. They also start analysing their own oeuvre and its place in their domain. Their metacognitive assessment takes into consideration considerable segments of previous work. Thus, eminent creators evaluate their own oeuvre not only from the point of view of their contemporaries, but also from the point of view of the long-term development of their domains and their place in the history of them. That is, they start to think about their intellectual legacy. Unfortunately, but for the information taken from autobiographical excerpts or case studies (Csikszentmihalyi 1996; Gardner 1993; Gruber and Wallace 2001), not much is known about the process of creative self-assessment among eminent creators. Table 1 summarizes how metacognition evolves at the four levels of creative development. 

## 5. Conclusions

I proposed here a framework to understand how spontaneous and controlled cognition impact creativity at different levels of creative development and during the generative and exploratory stages of creativity. Spontaneity is especially important in the early stages of creativity, but also in the activities based on improvisation. Generative processes in childhood are dominated by spontaneity. Schooling, on the other hand, plays a fundamental role in taming spontaneity, particularly mind wandering (Smallwood et al. 2007; Szpunar et al. 2013). At the highest levels of creativity, generation of ideas is disciplined, but at the same time creators develop the capacity to use their unproductive times generatively. The creative process is, then, not restricted to specific tasks or projects but extended in different networks of enterprise. Controlled cognition is especially relevant during the exploration of pre-inventive structures and becomes more influential as creativity develops.

Unfortunately, it is not known whether mindful mind wandering can be taught as a strategy to foster creativity. This issue is relevant because, as I have argued here, metacognition is a double-edged sword in creativity. It is detrimental in the generative stage, but it is essential in the exploratory stage. One way to overcome this dual impact of metacognition in creativity is through mindful mind wandering. Individuals learn when and how to let their mind wander and when and how to focus their attention on the task at hand. This is possible because creativity is, in real life, an extended process. The 10-year rule in the study of expertise and exceptional achievement (Hass and Weisberg 2015) as well as numerous case studies show that extraordinary achievement in creativity depends upon long-term endeavours (Gruber and Wallace 2001). Therefore, insight should also be studied in these prolonged initiatives. One of its attributes is its “two side past-closing/future opening structure” (Cosmelli and Preiss 2014, p. 50). While insight closes a gap in the past, it opens opportunities for the emergence of new problems. Mindful mind wandering not only keeps this dynamic going on, but also helps creators to define their entire identity as creative problem solvers. Charles Darwin’s invention of the theory of evolution is a canonical illustration of the role insight has in eminent creativity during a temporally extended process of exploration (Gruber and Wallace 2001). It is worth noting that the nature of this process evolves during the life of a creator. On the one hand, the development of expertise requires a high level of consciousness and controlled cognition, particularly when creators are learning their craft. On the other hand, exceptional achievement involves spontaneous cognition and openness to experience (Grosul and Feist 2014; McCrae and Greenberg 2014). The question remains about how mindful mind wandering interacts with mindless mind wandering in the most advanced levels of creativity.

To understand the relationship between cognitive flexibility and creativity, it is necessary to consider the role that the fluctuations between spontaneous and controlled cognition play in the creative process. The paper proposes that, as creators develop, they become more capable of monitoring and managing these fluctuations intentionally. This process involves second-order metacognition, that is, professional creators develop meta-strategies to identify when cognitive control is helpful to the creative process and when it is not. This process is easier to understand taking into consideration the multidimensional nature of metacognition. As noted, metacognitive experiences are more important in the generative stage of creativity, whereas metacognitive knowledge is more influential in the exploratory stages. In turn, metacognitive abilities are activated during both generation and exploration of pre-inventive structures by metacognitive knowledge and metacognitive experiences. Thus, while the generative process engages with metacognition through a bottom-up process driven by experiences, the exploratory process engages with metacognition trough a top-down process driven by knowledge and other mental models. 

A proper understanding of the relationships between cognitive flexibility, metacognition, and creativity might require going beyond the laboratory. That is, these processes must be studied in extended processes of creativity and not only through conventional experimental studies that are, too, localized in time (Cosmelli and Preiss 2014). As it is known, research on problem-solving has been commonly confined to laboratory situations. Thus, it is necessary to go beyond the laboratory to fully understand these creative phenomena. Specifically, initiatives of investigation that try to track mind wandering in real life (McVay et al. 2009) can be expanded to consider its relationship with creative problem-solving as well. By investigating attentional fluctuations in real life, we can generate new models of creative problem-solving with more ecological validity as well as explore problems that are not commonly addressed in traditional experiments. Furthermore, these initiatives will be instrumental for integrating two commonly disparate areas of creativity research: laboratory research of cognitive processes and real-life research of creative persons.

## Figures and Tables

**Table 1:** Mind wandering and metacognition in the development of a creative person.

Level	Activities	Cognition	Source of Evaluation	Focus of Evaluation
Mini-C	Exploration and improvisation	Spontaneous cognition	None or personal	Unique product
Little-C	Learning the rules	Spontaneous and controlled cognition	Peers and teachers	Samples of work
Pro-C	Developing expertise	Mindful mind wandering	Mentors and gatekeepers	Major work(s)
Big-C	Developing networks of enterprise	Extended reflection	Self and domain	Oeuvre

## Data Availability

Not applicable.
